# Emotion Detection for Social Robots Based on NLP Transformers and an Emotion Ontology

**DOI:** 10.3390/s21041322

**Published:** 2021-02-13

**Authors:** Wilfredo Graterol, Jose Diaz-Amado, Yudith Cardinale, Irvin Dongo, Edmundo Lopes-Silva, Cleia Santos-Libarino

**Affiliations:** 1 Departamento de Computación y Tecnología de la Información, Universidad Simón Bolívar, 1080 Caracas, Venezuela; 15-10639@usb.ve (W.G.); ycardinale@usb.ve (Y.C.); 2 Electrical and Electronics Engineering Department, Universidad Católica San Pablo, 04001 Arequipa, Peru; jose_diaz@ifba.edu.br; 3 Electrical Engineering, Instituto Federal da Bahia, 45078-300 Vitoria da Conquista, Brazil; edmundo_lopes@ifba.edu.br (E.L.-S.); cleialibarino@ifba.edu.br (C.S.-L.); 4 Estia Institute of Technology, University Bordeaux, 64210 Bidart, France

**Keywords:** social robots, natural language processing, ontology, emotion detection, text classification

## Abstract

For social robots, knowledge regarding human emotional states is an essential part of adapting their behavior or associating emotions to other entities. Robots gather the information from which emotion detection is processed via different media, such as text, speech, images, or videos. The multimedia content is then properly processed to recognize emotions/sentiments, for example, by analyzing faces and postures in images/videos based on machine learning techniques or by converting speech into text to perform emotion detection with natural language processing (NLP) techniques. Keeping this information in semantic repositories offers a wide range of possibilities for implementing smart applications. We propose a framework to allow social robots to detect emotions and to store this information in a semantic repository, based on EMONTO (an EMotion ONTOlogy), and in the first figure or table caption. Please define if appropriate. an ontology to represent emotions. As a proof-of-concept, we develop a first version of this framework focused on emotion detection in text, which can be obtained directly as text or by converting speech to text. We tested the implementation with a case study of tour-guide robots for museums that rely on a speech-to-text converter based on the Google Application Programming Interface (API) and a Python library, a neural network to label the emotions in texts based on NLP transformers, and EMONTO integrated with an ontology for museums; thus, it is possible to register the emotions that artworks produce in visitors. We evaluate the classification model, obtaining equivalent results compared with a state-of-the-art transformer-based model and with a clear roadmap for improvement.

## 1. Introduction

Nowadays, the presence of robots are becoming more common in human daily life [1]. Service robots concretely share environments with human beings to actively collaborate with them in specific daily tasks, such as serving as an assistant to nurses in patient walking and patient sitting tasks in hospital environments [2] or working as a student receptionist at a university [3]. Consequently, human–robot interactions (HRI) need to be revised in order to make the integration of robots into society as seamless and natural as possible.

An emerging approach to modeling social behaviors for service robots is based on emotion recognition. The detected emotion can be used for many purposes, such as dictating a robot’s behavior and accordingly adapting interactions with humans (in real-time) [4,5,6,7,8] or associating the emotion to events or objects in specific domains (e.g., opinions about commercial products, emotions produced by artworks in museums, and emotions produced by plates in restaurants) for further analysis [9,10]. From this perspective, keeping this information in semantic repositories offers a wide range of possibilities for automatically modeling the robot’s behavior or for supporting the implementation of smart applications.

The information from which emotion detection is processed can be gathered for robots in real-time via different media as they interact with humans, such as text, speeches, images, or videos [11]. This multimedia content is processed to recognize emotions/sentiments with proper techniques, for example, by analyzing faces and postures in images/videos based on machine learning or by converting speech into text to perform the emotion detection with well-known natural language processing (NLP) approaches [12,13,14,15].

In this context, we propose a framework to allow social robots to detect emotions and to store this information in an ontology-based repository. The detected emotions and other information are stored in a semantic repository based on an extensible ontology, called EMONTO (an EMotion ONTOlogy), to represent emotions. EMONTO can be extended with other specific domain ontologies representing entities with which the emotion can be related. The detected emotion can be used for many purposes, such as accordingly designing the reactions and actions of robots or combining the semantic information of emotions with other ontologies related to a robot’s tasks (e.g., SLAM (Simultaneous Localization And Mapping), navigation, and perception) [16] or with domain-specific ontologies related to the environment where robots work (e.g., museums, restaurants, and hospitals) [17].

To show the suitability of our approach, we develop a first version of this framework focused on emotion detection in text and tested it with a case study of tour-guide robots for museums. The robots receive the text directly from people or by converting speech to text. Then, we used NLP transformers to make sentence embeddings, transforming the text to a vector representation. Thus, the classification problem was solved in this new vector space, resulting in emotion labels for each text. The first version of the framework relies on (i) a speech-to-text converter based on the Google Application Programming Interface (API) and a Python library; (ii) a neural network to label the emotions in texts based on NLP transformers; and (iii) EMONTO, an ontology to represent emotions integrated with an ontology for museums; thus, it is possible to register the emotions that artworks produce in museum visitors for further analysis.

This first version of the framework offers the option for people to communicate with robots directly by text or for robots to perform an analysis on written sources to which they have access, such as social networks. In some situations, text may be the best or only way for people to communicate with robots; for example, in really loud or busy environments where the robots struggles to identify a single person or cannot properly hear voice commands, or for impaired people who cannot properly speak and rely on text messages to communicate their thoughts. Additionally, this approach can be incorporated in a chatbot to guide the responses according to the detected emotions.

We describe the stacked ensemble of transformer models to detect emotions from text and experimentally evaluate it. We obtain acceptable results with a clear roadmap for improvement, compared with the NVIDIA-AI [15], a state-of-the-art transformer-based model applied in the same task. The results show a micro F1 score of 0.6360, a macro F1 score of 0.4995, and a Jaccard index of 0.5076 overall for the model.

In summary, the contributions of this research are (i) a general framework for emotion recognition in social robots, considering all the sensing capabilities of robots; (ii) EMONTO, the ontology of emotions integrated to the framework that provides the base for implementing complex and smart applications from the detected emotions, related to the environment of the social robot; (iii) a first version of the framework, as a proof-of-concept, aimed at emotion recognition from text obtained directly or by converting sound to text; and (iv) an NLP transformer model for emotion recognition in text that behaves similar to a state-of-the-art approach.

We present our research organized as follows. Section 2 describes the related work about social robots and emotion detection from speech and from text, and the use of ontologies to store the recognized information. Our framework for emotion detection based on text analysis for social robots is detailed in Section 3. Section 4 presents EMONTO, an extensible ontology that represents emotions and supports the development of smart and interoperable applications. An experimental evaluation of the model to detect emotion from texts is presented in Section 5. We discuss the results obtained in Section 6. Finally, we conclude in Section 7.

## 2. Related Work

In this section, we first survey recent studies that show the importance of emotion recognition in improving HRI for social robots. Then, we describe some works in the field of emotion detection from text. Finally, we describe recent studies proposing approaches to recognizing emotion supported by ontologies in the context of social robotics. We highlight the relation to or difference of the cited works from our research on these three aspects.

### 2.1. Social Robots and Emotion Detection from Speech

Nowadays, emotion recognition is essential to improving HRI, in particular, for service robots. Emotion recognition allows us to model the social behavior of robots that share spaces with humans, thus making social robots a growing area of robotics where psychology and sociology aspects converge [18]. However, the design and development of robot’s abilities in acting and interacting physically, emotionally, socially, and safely with humans has generally been poorly understood so far [19]. Indeed, in order to socially interact with humans, a robotic system should be able not only to understand user behavior and intentions but also to estimate their emotional state with a high level of acceptability and usability [9]. Thus, much more attention should be paid to an assessment of the feasibility of a social robot in real-life scenarios among different cultures and people [4].

Thus, a growing effort is shown in recent works for natural and seamless integration of robots into society, supported by emotion recognition to model their social behaviors. The sensing capabilities of robots allows us to gather several multimedia content, from which emotions can be recognized. Although our first version of the framework considers only voice and text, in the future, we aim to implement the capacity to process all other content. For now, we revise some works dealing with emotion recognition in speech and text.

In [5], openSMILE, a framework to extract several speech emotion features was described; it was based on fuzzy C-means to cluster the training set into multiple sub-classes; then, multiple random forest methods make the decision to identify the emotion (i.e., angry, fear, happy, neutral, sad, and surprise) from the selected speech features. In [6], a multi-modal emotion detection model for personal assistant robots was presented. This model was implemented by means of emotion recognition from voice and images, and both outputs were merged to achieve the detected emotion using machine learning techniques. A speech emotion recognition (SER) system based on a combinations of convolution neural network (CNN) and random forest models was presented in [7]. The CNN model was used to extract speech emotion features from the normalized spectogram, and the random forest algorithm performed the classification. SER was used on a NAO robot with recorded sound commands, working with four basic emotions: happiness, anger, sadness, and joy.

A behavior modulation system (BeMoSys) for social robots was proposed in [8]. BeMoSys provides robots with the capacity for emotional speech recognition based on signal-processing and machine learning techniques to perform the emotion classification. It considered five inputs corresponding to the five emotions extracted from the speech signal in order to derive the estimated level of happiness. According to this value, the appropriate robot behavior was set.

We found few recent works that combine speech and text in the context of robotics for emotion recognition. In [20], a system that combines the analysis of prosodic features of the speech and sentiment analysis on the text (obtained from the speech) to recognize emotion and let robots properly express reactive emotions was presented. Sentiment analysis over the text allows for the identification of the polarity (i.e., negative, neutral, and positive), for which the value (−1, 0, or 1) is used in emotion classification, combined with an analysis of the prosodic features. The considered categories in this study were embarrassment, unsettling, noticing, remembering, unexpectedness, surprise, hesitation, anxiety, pain, dislike, disappointment, pleasure, and anger.

These recent works demonstrate the current trend of voice analysis for emotion recognition in social robots in improving HRI and modeling social behaviors for robots. However, there are still limitations on the quality and precision of the results; thus, more accuracy models should be used, such as text analysis.

With our proposed framework, social robots are able to recognize emotions in humans with whom they interact and accordingly to adapt their behaviors and interactions. Hence, our work is a contribution to social robotics. Besides that, converting voice into text represents more benefits is social robotics: robots can analyze writing sources (e.g., social media) as an alternative for speech-impaired people when communicating with robots, does not neglect other multimedia sources, and can be used to improve chatbot applications.

### 2.2. Emotion Detection from Text

**Figure 1:**
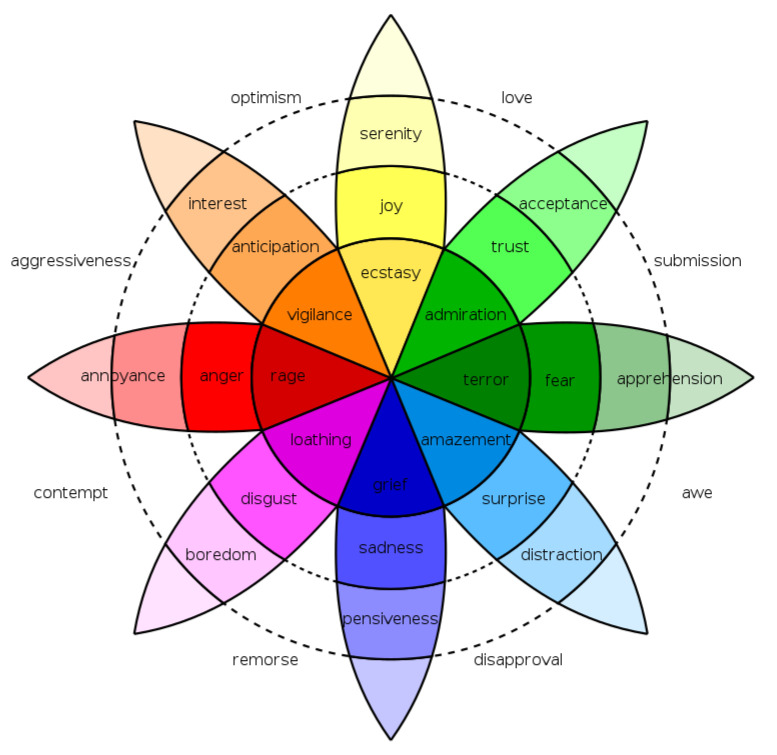
An extract of Plutchik’s wheel of emotions.

For emotion recognition systems, an important aspect to consider is the emotion models that delimit the classification process [13]. While according to Cowie [21], in the field of psychology, there are many different theories regarding the representation of emotions, within the area of NLP, there are two that stand out as the most used: Ekman’s basic emotions [22] and Plutchik’s wheel of emotions [23]. Ekman’s model consists of six basic emotions: anger, disgust, fear, happiness, sadness, and surprise. Plutchik’s model consists of a multi-dimensional approach, where there are four opposing pairs of axes and emotions are defined as different points along these axis. In this model, an emotion is determined by an emotion axis and its intensity. These axis pairs are joy–sadness, anger–fear, trust–disgust, and surprise–anticipation. Figure 1 shows an extraction of the Plutchik’s model, with these axis and intensity denoted with colors in the concentric circles; from these emotions, many other emotions can be derived as a combination of other emotions and intensities. Most works in emotion detection consider a small subset of these group of emotions.

Most common approaches to performing emotion classification and general NLP tasks are based on recurrent neural networks (RNN) or some variants of them, such as long short-term memory (LSTM) [24], multiplicative LSTM (mLSTM) [25], or gated recurrent unit (GRU) [26,27,28]. However, these approaches suffer from a couple of related issues: the complex nature of emotion expression and the shortage of quality data for this task [14]. Finding quality label data for this task is hard to do, at least in order to completely train these types of models. Nevertheless, these limitations have been overcome by an attention-based transformers architecture [29], which is inspired by the encoder–decoder architecture used for sequence to sequence (Seq2seq) tasks. In high-level terms, it consists of two stacks (one of encoders and one of decoders), where the encoder and decoder use some form of self-attention (https://jalammar.github.io/illustrated-transformer/ (accessed on 18 December 2020)) and a feed forward neural network. Each encoder in the stack receives its input from the previous layer, except the first one that receives a series of tokens. Then, the final encoder in the stack sends its output to every decoder. Then, each decoder uses this and the input from the previous layer in the stack to return its output. Afterward, the output for the final decoder is passed to a linear + softmax function to finally return the output of the network. Recently, it has become more and more common to use transformers for NLP in texts, since they have been proven to be effective in many different tasks (e.g., masked token prediction, next sentence prediction, question answering, machine translation, summarization, natural language inference, and sentiment analysis) [30,31,32]. This is thanks to the fact that they are trained with unsupervised learning models, diminishing the need for labeled data. Transformers are very effective in transfer learning, allowing researchers to pretrain these transformers with large amounts of general-purpose texts and then to finetune these models for their specific tasks with good results, less effort, and labeled data, such as NVIDIA-AI (currently named Megatron-LM), the model proposed in [15], by researchers of NVIDIA.

For the classification task, the transformer architecture allows us to test different types of methods to approach it. An ensemble is a successful approach in reducing the variance of sub-models by training multiple sub-models to combine the predictions from them [33]. There are many ways to implement an ensemble (e.g., stacking, boosting, and bagging). Recently, many experiments were conducted with ensemble neural networks using transformers. For example, boosting bidirectional encoder representations from transformers (BERT) proposed in [33] is a model to introduce the multi-class boosting into BERT [32]; the classifier for detecting aggression in social media posts by bagging BERT models presented in [34]; and a stacked ensemble with 25 BERT to classify fake news, described in [35].

Currently, there are many available implementations of these transformers, such as the *Hugging Face Transformers* library [36], which provides a wide variety of implemented pretrained transformers that work with a classic NLP workflow; the *Simple Transformers* library (https://github.com/ThilinaRajapakse/simpletransformers (accessed on 18 December 2020)), which is a simplification of the previous one, suitable for non-expert researchers; and the *Sentence Transformers* library [37].

Our classification model is based on a transformer architecture, supported on the *Simple Transformers* library.

### 2.3. Social Robots, Emotion Detection, and Ontologies

In the domain of social robots and emotion detection, ontologies have been applied in order to represent the emotional knowledge. The authors of [38] proposed a cognitive architecture for an emotion-aware robotic system by using a multilayer perceptron neural network and several features associated with facial expression (e.g., brows, lips, and face muscles motions) and emotions on speech (e.g., words used, syntactic structure, and meaning). They used an upper ontology to cover all common-sense concepts of human states and robotic systems (EmUO) and *HTemp* ontology to represent the complex context using its n-ary schema. In [39], OntCog that is based on IEEE 1872-2015—IEEE Standard Ontologies for Robotics and Automation, was proposed. The main concept in OntCog was RobotSense that is related to all concepts in this ontology. Other properties such as IRI_Emotion to associate an emotion to a smell were also defined. The same authors propose in [40] an architecture, called cognitive model development environment (CMDE), to capture and process data collected by sensors present in the robot and to store the perception of the environment using an ontology. This ontology is the one proposed in [39]. Other works have used ontologies to store information in the context of robots but for activity detection [41], user-intention recognition [42], object detection [43], etc.

These studies used semantic knowledge representation in different aspects of emotion sensing for social robots; however, the ontologies proposed neither represent emotions nor are extensible to be able to relate such emotions to robots’ tasks or to other entities. We propose EMONTO, an extensible ontology of emotions, and we incorporate it into the framework; thus, this semantic information can be used for many proposes in the context of social robotics.

## 3. A Framework for Emotion Detection for Social Robots

**Figure 2:**
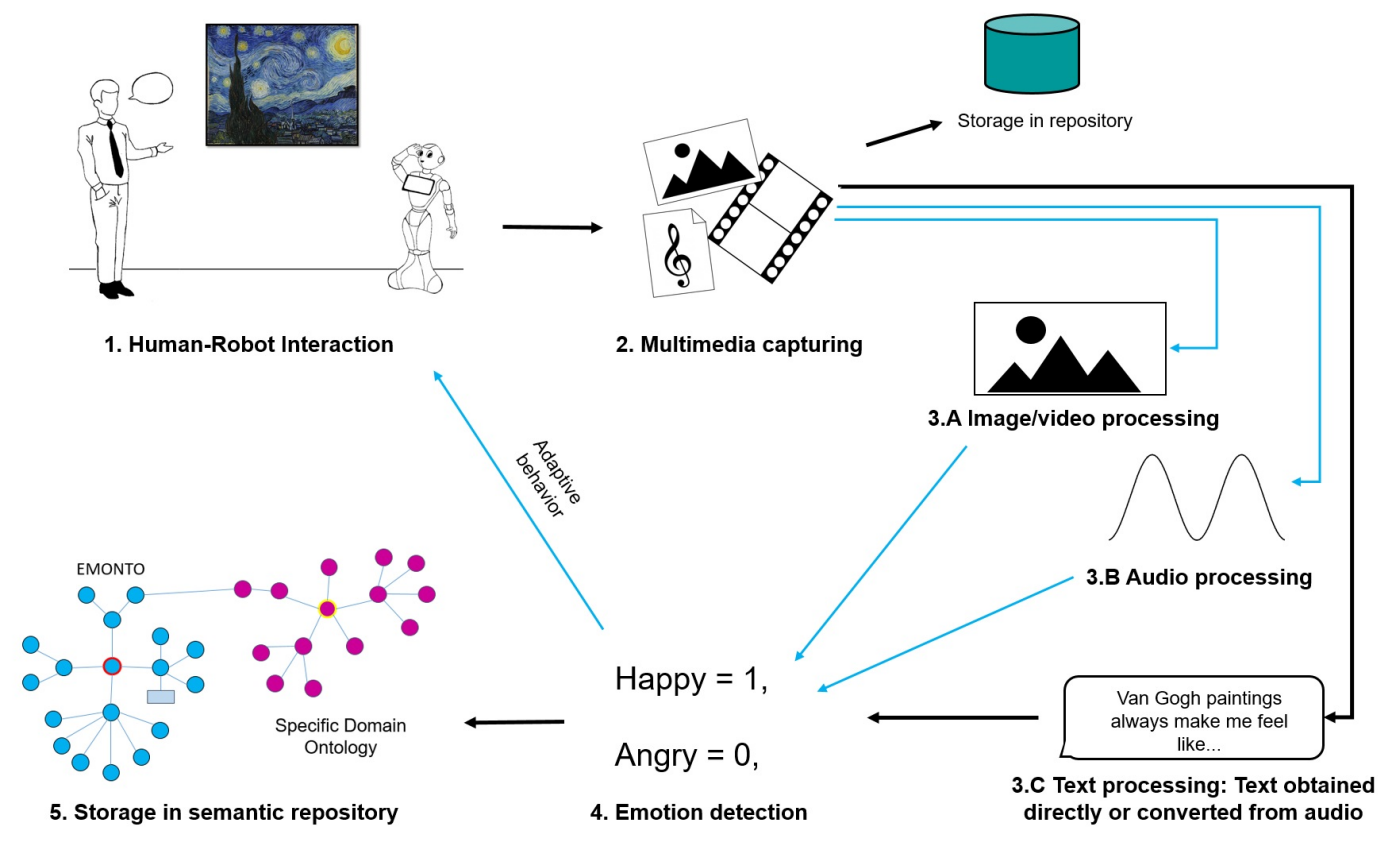
General overview of the framework.

The proposed framework is a process divided into five sequential steps, as shown in Figure 2: (1) *human–robot interaction*; (2) *multimedia capturing*; (3) *multimedia processing*: *image/video processing, audio processing*, and *text processing* (text obtained directly or converted from audio); (4) *emotion detection*; and (5) *storage in a semantic repository*. From the human–robot interaction (step 1 in Figure 2), multimedia content is gathered from the robot’s sensory capacity; these raw data are saved in a repository (step 2 in Figure 2). The following steps imply transformations of the obtained data from the previous step, starting with the conversion of raw input obtained from the user to proper representations for emotion analysis (e.g., feature vectors and text) (step 3 and step 4 in Figure 2) and finishing with the population of an emotion ontology that can be used for different purposes (step 5 in Figure 2).

This framework can be instantiated in social robots systems, taking advantage of all the sensing capacities of robots. As a proof-of-concept, in this work, we implement the whole pipeline taking into account only text and sound for a service robot in a museum getting feedback from users regarding the artworks. In this section, we detail the steps related to emotion recognition for this first implementation of our proposed framework. The next section presents EMONTO and its instantiation.

### 3.1. Human–Robot Interaction

In this phase, the social robot interacts with people in the museum, starting conversations with a neutral tone, but eventually, after engaging with the person or group of people, it can adjust its tone to act according to the detected emotions (e.g., excited, energetic, and comprehensive).

The robot must attempt to get, via a series of questions, the visitor’s opinion regarding the artworks; name; and, if possible, some unique identifier such as a social media handle. With these data, it is possible to identify registered/frequent visitors (possible facial and posture recognition if images and videos are considered); thus, the robot can remember them and choose how to initiate contact or a different type of conversation. Moreover, data mining in social media is also possible in determining users’ profiles. According to the detected emotion in step 4 (see Figure 2), the robot can adapt its behavior (e.g., change the tone to fit the mood of the user and decide whether to continue the conversation).

### 3.2. Multimedia Capturing

During the interaction with the visitor, the robot records and saves all multimedia content captured by its sensors. For the case of speech, the robot keeps in the audio files all answers and comments it receives from the user. Although, the emotion is detected from the extracted information from the multimedia raw data, keeping the original data (e.g., images, videos, and audio) permits additional analysis, for example to extract prosody features from speech or to extract context information from images, for further experiments.

For the first version of our framework, in this step, we implemented *speech capturing* with an ambient noise reduction algorithm to record audio and for speech-to-text conversion in order to reduce noises that can interfere with the results. According to the sensors’ capabilities, robots can also gather images and videos and can use facial and body posture recognition to extract the related features for emotion detection.

### 3.3. Multimedia Processing

In this step, the robot converts the multimedia raw data into proper representations for emotion analysis. For example, feature vectors from images and videos, normalized spectograms from audio, and text from speech.

For the first version of our framework, in this step, the robots can receive text directly or by converting audio to a text format. We developed a speech-to-text algorithm using the available Google API [44] and the SpeechRecognition Python library, which is specialized in speech processing, audio reading, and noise reduction, based on a Hidden Markov Model for speech recognition and an RNN to tackle the sequence problems, where the timing is variable.

For the case of images and videos, features extracted can obey traditional machine learning models as well as more complex models such as multimodal-based ones that are becoming popular for emotion recognition in images and videos. We plan to incorporate such techniques to improve the implementation of our framework.

### 3.4. Emotion Detection

According to the multimedia data considered, a wide range of emotion detection techniques can be implemented, mostly based on machine learning models.

For the first version of our framework, in this step, we approached the emotion detection task as a multi-label classification problem, modeled with a Transformer architecture and meta-learning. We considered eleven emotions: *anger, anticipation, disgust, fear, joy, love, optimism, pessimism, sadness, surprise, and trust*. The classification task consisted of labeling a text as “neutral or no emotion” or as one or more of the previously mentioned emotions, i.e., a binary vector indicating if each emotion was detected (1) or not (0).

**Figure 3:**
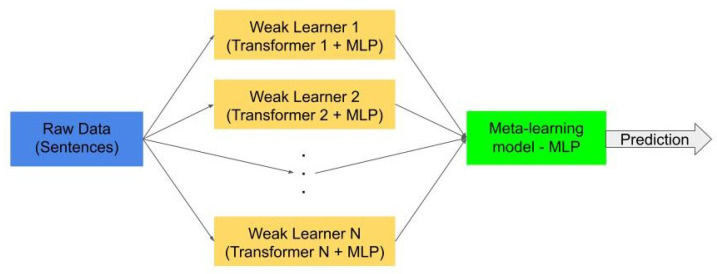
Stacked ensemble architecture implemented.

**Figure 4:**
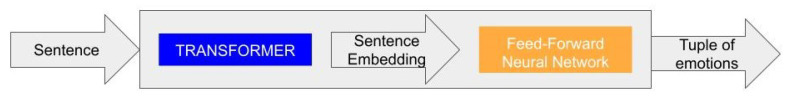
Structure of each weak learner in our model.

Based on the available pretrained transformers, we designed a stacked ensemble architecture, as shown in Figure 3. Each weak learner classifier consists of a transformer, used to create a sentence embedding and a Multi-Layer Perceptron (MLP), with two hidden layers of sizes 500 and 300 (see Figure 4). To avoid overfitting of our MLP, we used an early stop in the training, using 10% of the training data to validate in each iteration if the training is still effective. For implementation of the transformers, we used the Sentence Transformers library (https://github.com/UKPLab/sentence-transformers (accessed on 18 December 2020)) [37], since they are specifically pretrained transformers to perform semantic textual similarity tasks, leading to sentence embeddings. This is a mapping of sentences to a vector space such that sentences with similar semantic meanings are close in this new space. From the Sentence Transformers library, we used the *roberta-large-nli-stsb-mean-tokens*, *distilbert-base-nli-mean-tokens*, and *bert-large-nli-stsb-mean-tokens* transformers to perform the sentence embeddings. Therefore, we use a stacked ensemble with three different weak learners.

The meta-learning consists of an MLP with one hidden layer of 10 neurons. All MLPs were implemented using sklearn (https://scikit-learn.org/stable/index.html (accessed on 18 December 2020)) [45] and using the default adam solver, the cross-entropy loss function, the RELU activation function, a regularization term set at 0.0001, and a random_state initialized in 1.

Previously, word embedding was used for emotion detection in text and for the general NLP task [14]. One common approach to processing a given sentence is to tokenize the sentence with word embeddings and to then pass the sequence of tokens to a RNN. However, with transformers, we have a simple way to directly create embeddings for whole sentences.

## 4. Emotion Ontology and Instantiation

Data provided by the recognition methods are normally stored for analysis, which can generate a complex network of knowledge. The ontologies are well-defined and standard models to represent the knowledge managed by these applications. In this work, we propose EMONTO, an ontology to represent emotions and to support the development of smart and interoperable applications.

### 4.1. EMONTO: An Extensible Emotion Ontology

**Figure 5:**
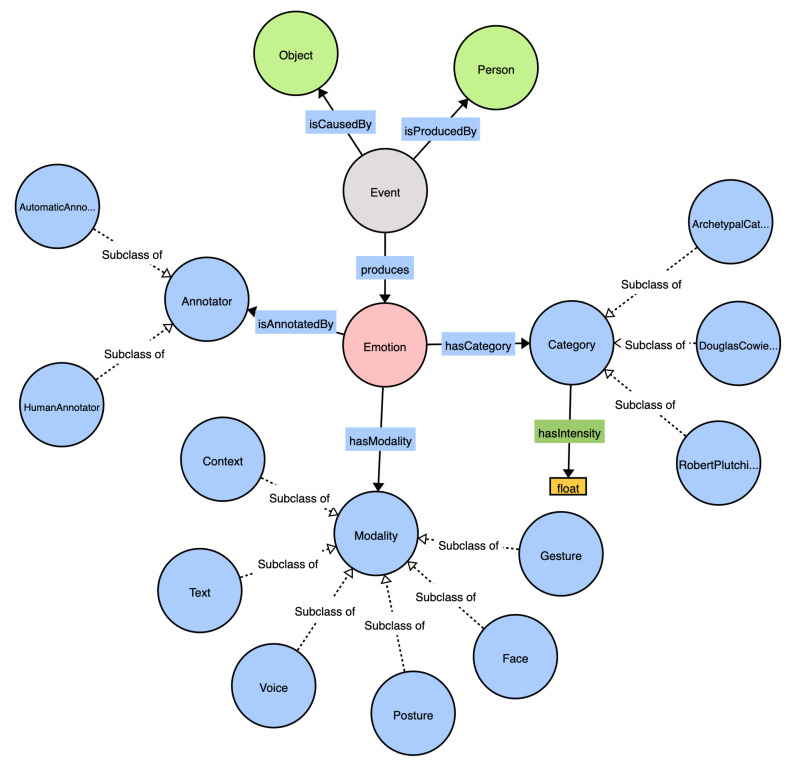
EMONTO: an extensible emotion ontology.

EMONTO is an extensible ontology that represents emotions under different categorization proposals. We adopted some concepts from several emotion ontologies [46,47] to provide compatibility. Figure 5 shows the main classes of EMONTO. The central class is emo:Emotion, which has a category (hasCategory) according to a Category class. The current version of EMONTO considers archetypal [46], Douglas Cowie [46], and Robert Plutchik [48] emotion categorizations, which group emotions into 6, 25, and 56 (8 basic emotions) values, respectively. Nevertheless, any other categorization model can be integrated as a subclass of emo:Category.

EMONTO has emo:Event as a class that connects the emo:Object, emo:Person, and emo:Emotion entities. An emotional *Event* is produced by (isProducedBy) a *Person* and is caused by (isCausedBy) an *Object* (e.g., artworks, candidates, and plates). An *Event* can produce several *Emotions*. The entities emo:Object and emo:Person are general classes that can connect other ontologies, such as museum and artwork ontologies [17] as *Object* or user-profile ontologies [49] as *Person*. The idea is to make EMONTO extensible and flexible to be easily adopted in scenarios where data related to the recognized emotions need to be stored for further analysis. The ontology provides the modality (emo:Modality) of the information used to recognize the emotion (e.g., emo:Gesture, emo:Face, emo:Posture), and the type of annotator (emo:AutomaticAnnotator and emo:HumanAnnotaton). Moreover, a datatype property emo:hasIntensity is associated to the category to express the level of confidence (a float value between 0.0 and 1.0).

### 4.2. Instantiation of our Emotion Ontology

Once the emotions are recognized by our multi-modal recognition method, an *Event* is created to represent the emotional event (<E0001 rdf:type emo:Event>), associated to some datatype properties (e.g., <E0001 emo:createdAt 1607984678>). Then, the *Event* is associated to either new instances of *Person* and *Object* (<E0001 emo:isProducedBy P0001> and <0001 emo:isCausedBy O0001>) or to existing values. *Person* and *Object* should be also recognized by using other models that can be combined with our multi-modal method in the proper data acquisition phase. For example, by applying facial recognition to identify registered or new users in the system and object detection to recognize specific objects (e.g., artworks in museums and plates in restaurants). This work is focused on emotion recognition; *Person* and *Object* detection is beyond the scope of this research.

According to the results of the emotion recognition method, one or more emotion entities are created (<EM0001 rdf:type emo:Emotion>, <EM0002 rdf:type emo:Emotion>, <EM0003 rdf:type emo:Emotion>, etc.) and associated to the *Event* (<E0001 emo:produces EM0001>, <E0001 emo:produces EM0002>, <E0001 emo:produces EM0003>). Each emotion has a *category* (<EM0001 emo:hasCategory C0001>, and <EM0002 emo:hasCategory C0002>, <EM0003 emo:hasCategory C0003>) which can be archetypal, Douglas Cowie, or Robert Plutchik classifications (<C0001 rdf:type ArchetypalCategory>, <C0002 rdf:type ArchetypalCategory>, and <C0003 rdf:type ArchetypalCategory>). The datatype properties emo:hasIntensity and emo:emotionValue are added to the *category* (<C0001 emo:hasIntensity 0.13> and <C0001 emo:emotionValue "anger">) with the values obtained by the emotion recognition method (e.g., *0.13* as intensity and *“anger”* as emotion). *Modality* and *annotator* are also instantiated. A pseudocode of creating triples is shown in Algorithm 1. First, libraries related to Resource Description Framework (RDF) management have to be import (line 1), then a new graph, which contains the RDF triples, is created (line 2). Namespaces of the ontology are added (lines 3–5). From line 6 to line 17, new RDF triples are added to the graph.
 **Algorithm 1:** Creating RDF triples. 1 import RDF libraries 2 g = Graph() 3 EMO = Namespace(“http://www.emonto.org/ (accessed on 18 December 2020)”)        //Creating a Namespace. 4 g.add_namespace(“emo”, EMO)        //Adding the namespace EMO. 5 g.add_namespace(“foaf”, FOAF)        //Adding the namespace FOAF, which is already defined in the libraries. 6 g.add_triple((EMO.E0001, RDF.type, EMO.Event))        //Creating an Event. 7 g.add_triple((EMO.P0001, RDF.type, FOAF.Person))        //Creating a Person. 8 g.add_triple((EMO.O0001, RDF.type, EMO.Object))        //Creating a Object. 9 g.add_triple((EMO.EM0001, RDF.type, EMO.Emotion))        //Creating an Emotion 1. 10 g.add_triple((EMO.EM0002, RDF.type, EMO.Emotion))        //Creating an Emotion 2. 11 g.add_triple((EMO.EM0003, RDF.type, EMO.Emotion))        //Creating an Emotion 3. 12 g.add_triple((EMO.E0001, EMO.produces, EMO.EM0001))    //Associating E0001 to EM001. 13 g.add_triple((EMO.E0001, EMO.produces, EMO.EM0002))    //Associating E0001 to EM002. 14 g.add_triple((EMO.E0001, EMO.produces, EMO.EM0003))    //Associating E0001 to EM003. 15 ... 16 g.add_triple((EMO.C0001, EMO.hasIntensity, Literal(0.13)))          //Intensity value. 17 g.add_triple((EMO.C0001, EMO.emotionValue, Literal(“anger”)))    //Recognized emotion. 18 ...


### 4.3. Querying Our Emotion Ontology

The semantic repository enables the possibility of implementing simple and advanced queries in order to retrieve specific values or to infer new knowledge, in both cases for data analysis. For example, a simple query can retrieve information about the emotions produced by a specific artwork. The pseudocode presented in Algorithm 2 retrieves the emotions produced by the artwork The_Starry_Night: in line 1, libraries related to the RDF manipulation are imported; then, a graph should be initialized to read the RDF triples (lines 2 and 3); the namespaces used in the ontology have to be added (line 4); and finally, following the SPARQL syntax, a query is performed (lines 5–15).
 **Algorithm 2:** Obtaining the artwork The_Starry_Night. 1 import RDF libraries 2 g = Graph() 3 g.read(“database_emotions.ttl”, format=“ttl”) 4 g.add_namespace(“emo”, “http://www.emonto.org/ (accessed on 18 December 2020)”) 5 qres = g.query(“SELECT ?emotion_l 6            WHERE { 7            ?emotion a emo:Emotion ; 8            ?emotion emo:emotionValue 9            ?emotion_l . 10          ?event a emo:Event ; 11          ?event emo:produces ?emotion ; 12          ?event emo:isCausedBy 13          ?object. 14          ?object a emo:Object ; 15          ?object rdfs:label “The_Starry_Night” . 16 }”)

Other information such as the artworks that produce the emotion “surprise” can be also obtained (see Algorithm 3).
 **Algorithm 3:** Obtaining the emotion surprise. 1 “SELECT ?object_l 2             WHERE  3            ?emotion a emo:Emotion ; 4            ?emotion emo:emotionValue “surprise”. 5            ?event a emo:Event ; 6            ?event emo:produces ?emotion ; 7            ?event emo:isCausedBy ?object. 8            ?object a emo:Object ; 9            ?object rdfs:label ?object_l . 10 ”

In these examples, the *Object* entity is an artwork, but an object could also be plates in restaurants, movies, or any entity that produce emotions. The extensibility of EMONTO allows researchers to integrate any specific domain ontology.

## 5. Experimental Evaluation of the NLP Transformer Model

In this section, we show the performance of our transformer-based method compared with the NVIDIA-AI model [15], which is also based on a transformer architecture. We detail the procedures for obtaining adequate data for training and testing, some important implementation details, and the comparative results.

We used the dataset for SemEval-2018 Task 1 [28], specifically the dataset E-c. This dataset contains tweets in English, each with its own ID and contents, that are labeled with a vector of eleven binary numbers, corresponding to the eleven considered emotions (*anger, anticipation, disgust, fear, joy, love, optimism, pessimism, sadness, surprise, and trust*). If the value at position *k* is 1, the tweet contains the emotion *k*; otherwise, the value is 0. Table 1 illustrates some samples taken from the dataset in which the first text presents the emotions *anger, fear*, and *sadness*.

Training of the model was performed with the *2018-E-c-En-train* file that contains 6838 tweets labeled as indicated previously, and all the result metrics were calculated using *2018-E-c-En-test-gold* that contains 3259 tweets. Training was performed using all 6838 samples of the *2018-E-c-En-train* dataset, since validation was done on the *2018-E-c-En-test-gold* dataset; thus, there was no need to split the data used for training. In Table 2, we present the number of positive values per emotion among the samples of the dataset; there is some class imbalance as the *surprise* and *trust* labels are poorly represented. However, since this is a multi-label classification, this is not a trivial issue to handle and we ended up finding better results without upsampling the data.

For metrics, as per the official competition rules, we used the Jaccard index to measure accuracy as this is a multi-label classification task, based on Equation (1), where *T* is the set of tweets in the dataset and where, for any given tweet *t*, $G_{t}$ is the set or the expected labels for *t* and $P_{t}$ is the set of predicted labels for *t*.

(1) $$Accuracy=\frac{1}{\left|T\right|}\sum_{t\in T}\frac{\left|G_{t}\cap P_{t}\right|}{\left|G_{t}\cup P_{t}\right|}$$

Algorithm 4 shows the general process of training: the implementations needed for both the transformers and the MLPs are imported (line 1 and line 2, respectively); they can be either user-made libraries or publicly available ones; then, the training samples as well as their expected labels are loaded (line 4 and line 5); the transformer models that will be used for each of the weak learners are also loaded (line 7); afterward, the training of the weak learners is done (lines 8–15); once the training cycle is performed, all predictions made by the weak learners are combined (line 17) to be used to train the meta-learner MLP; and this final classifier is created (line 19) and trained (line 20). This algorithm is available in Python (https://colab.research.google.com/drive/16Ja5MUFb_A0RmaBVKvcjYxGSmvmSTtMN?usp=sharing (accessed on 18 December 2020)).
 **Algorithm 4:** Training process.
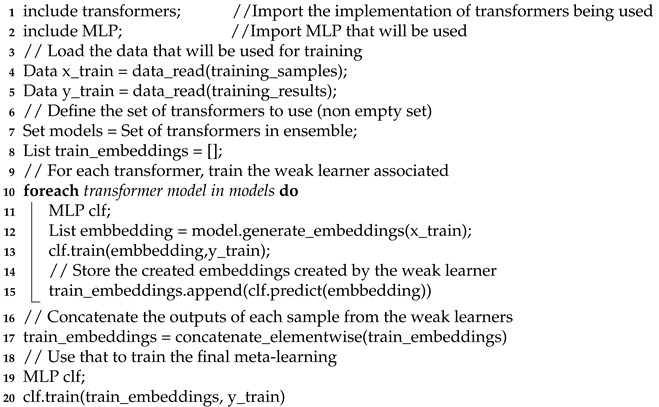


**Figure 6:**
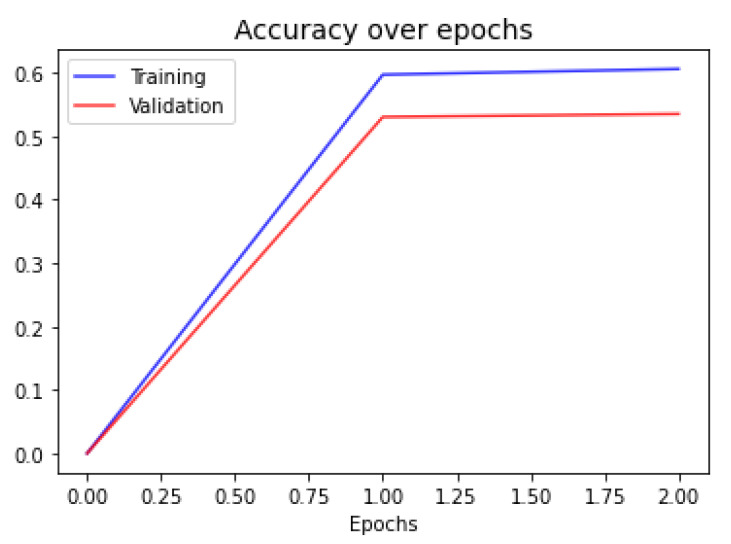
Validation curves for the final meta-learning.

In Figure 6, we show the accuracy evolution plot of the final meta-learning. This network finished training with only two epochs, which is expected given the difference in size of the number of free parameters in the network and the size of the training size.

Table 3 shows the obtained results using the sklearn built-in functions. We include the results of F1 score, precision, and recall from our model, both the micro average (i.e., calculating the number of positives and negatives globally among all classes) and the macro average (i.e., calculating the metric for each label and then finding their unweighted mean (https://scikit-learn.org/stable/modules/generated/sklearn.metrics.f1_score.html) (accessed on 18 December 2020)). The best results are in precision, which means that the model is good at avoiding false positives. The F1 scores show an overall ability for detecting true positives, up to 76%. However, it struggles when it comes to accurately detecting negative values, as shown by the recall scores. This statement is further confirmed by looking at the results of the confusion matrix in Table 4, that shows that the amount of false positives produced is consistently lower than the number of false negatives produced. The relatively big difference between macro and micro scores may be caused by a class imbalance in our training set. Table 2 shows that the labels *surprise* and *trust* have around one tenth of the samples that the most represented one (i.e., *love*) has. Thus, the values from the confusion matrix in Table 4 show that these poorly represented labels are the ones with the least amount of positive labels predicted, meaning that our model is not learning any pattern to classify them and is labeling most of the samples as not containing those emotions. Accuracy on the other hand seems to indicate that our model, on average, strays around 47% from the expected emotions per instance.

Compared with the NVIDIA-AI model, the results presented in Table 5 show that our model is behind the other by at most 7% across all evaluated metrics. This leaves us at a good starting point as. Unlike the NVIDIA-AI model which was fine tuned for the tweet dataset, fine-tuning our Transformers for this task and possibly changing our MLP classifiers for more refined models could lead to better results. We explore these possibilities further in the Discussion section.

## 6. Discussion

The first version of our framework, as a proof-of-concept, demonstrates the feasibility and suitability of a robot system able to recognize emotions from interactions with humans, which can be used to adapt the robot’s behavior and to instantiate a semantic repository, for further analysis. This experience also gives the opportunity to extract its current limitations and some lessons learned.

### 6.1. Improvements for the Classification Model

The classification method can be improved in several aspects. One of them is in the transformers. The ones we used were fine-tuned for semantic textual similarity of tweets, in which a lot of emojis and abbreviations are present. Conversations in museums are quite different; thus, a more appropriate dataset, with more appropriate texts, can be used to fine-tune the transformers.

As explained in Section 5, an imbalance in the dataset generates some problems when it came to training the model, as some emotions could not be correctly detected. While in single-label classification problems, there have been many techniques developed to mitigate this, in the multi-label case, there are not many solutions available to solve this. For example, one common technique in the single-label case is to randomly upsample the data. In the multi-label case, doing this could generate more instances of the other labels, generating even more imbalance. However, there have been some algorithms developed in recent years to approach this problem that were not used in this instance of our framework, such as multilabel synthetic minority over-sampling technique (MLSMOTE), that we plan to try in the future.

The ensemble method and the classifiers used for the weak learners could be also improved. As mentioned before, both boosting and bagging are other ensemble methods that have shown good performance in other tasks when used with transformers [33,34]; thus, it would be worth evaluating these methods for our model. We used MLP for the classifiers, as they tend to perform well in some classification tasks and are easy to use for multi-label classification. Nevertheless, there have been works attempting to classify sentences with other architectures, such as CNNs [50]; hence, this is another part of the current model that could be tweaked.

### 6.2. Extending the Framework’s Functionalities

Most social robots have the ability to record video and to capture images, along with the ability to record audio. Hence, the framework can be extended to be able to perform a multi-modal classification, considering different modalities, such as text, voice, face expressions, and body postures and gestures. Although this modification implies the design of a more complex classification model, the results would be improved and offer a wide range of applicability in the area of social robotics. As developed, our framework allows for a straightforward way to add different media to the learning process, as it would be a matter of simply creating a classifier for it and adding it to the stack, making it simple to continue development on it.

This framework is mainly useful for two purposes: enhancing social behavior for service robots and collecting data for more complex analysis. The ontological repository enables enlarging the possibilities to improve both purposes. It can be used as direct feedback to record internal statistics of what emotions are evoked by entities (e.g., pieces of art in the museum, plates in a restaurant, and candidates in elections). That way, the robot can adjust its tone and interactions to fit the mood according to the specific entity, instead of using a default tone.

Moreover, the capability of social robots in analyzing texts enable the opportunity to analyze social media to infer what types of people come to the place and what types of stimuli they are attracted to. Thus, with the gathered data and the inference capability of ontologies, the knowledge can be used for decision making. For example, in the museum case, we can obtain a clear rating of artworks, which can help the museum managers decide what pieces to display at any given moment or to decide how to dispose exhibitions.

As it is, this version of our framework can complement chatbot systems by guiding responses according to the detected emotion.

### 6.3. HRI Improved with Emotion Detection

In some scenarios for service robots, they have to choose the person to interact with and start the interaction; thus, a selection algorithm is needed. We suggest a selection algorithm tending to choose people that are alone or in “small" groups (“small", in this case is implementation dependent), since this makes it easier for social robots to attract their attention. Selecting a small group of people should also help the speech-to-text component of the framework as it would mean less chatter noise around the robot when it records the person’s speech.

Once the person or group of persons is selected, the robot should try to start a conversation. If the robot fails to engage a conversation with the user because, for example, the user ignored it, then it can repeat this step until it finds a person to engage with. Once this happens, a conversation is started. Initially, the robot should start with a neutral tone for all engaged persons, but once it has obtained enough data regarding the emotions people have within its context, it can adjust itself to make the interaction as natural as possible. For example, suppose this framework is implemented in social robots in an amusement park that has a roller coaster and a haunted house. Initially, all robots would start with neutral tones, but eventually, after engaging with many excited people, the ones on the roller coaster, they will adjust their tone to act excited and energetic as well. Meanwhile, after engaging with many scared people, the ones in the haunted house, they should start to act afraid as well.

### 6.4. EMONTO Extensibility

The output of the classification process strongly depends on the emotion representation model. For example, Ekman’s basic emotions model [22] supports a one versus all classification problem, while Plutchik’s wheel of emotions [23] implies a multi-label classification problem for a subset of possible emotions or a polarity problem among each of the axes of the wheel. EMONTO supports both approaches and can be extended with other emotion models without affecting the implementation of the framework. Moreover, the entities *Person* and *Object* of EMONTO can be replaced by entities defined in user-profile ontologies for *Person* and specific domain ontologies representing entities to which the emotions can be associated (e.g., artworks, museums, and dish) for *Object*. Moreover, EMONTO can be extended with other ontologies that model the specific task of the service robots (e.g., SLAM ontologies). Hence, EMONTO is flexible and extensible for any scenario in social robotics.

## 7. Conclusions

The need for interaction between machines and humans is becoming more common in people’s daily lives. The effort to improve these relationships through the interpretation of social behaviors are more and more frequent among research developed in the area of social robotics. The interpretation of the feelings of a human being is an important tool that a robot must know how to use to enact correct behavior in a social environment. Our framework is a clear contribution in this area, since it allows robots to interpret a person’s basic feelings and emotion recognition algorithms. Besides that, the information gathered by the robots and the outputs of the classification process can finally be organized in an ontology, leading to more complex analyses and uses of the data. The results obtained with a first implementation of our framework, as a proof-of-concept, show that the integration of speech-to-text and emotion detection algorithms with an ontology was a success despite the fact that there is still the possibility of being improved.

Our future research is focused on improvements of the framework, starting with the suggestions from Section 6 to provide a robust architecture in the community to develop third-party applications in social robotics. We realize the need to incorporate more interpretation characteristics that can complement the detection of a person’s feelings in our framework, such as detection of the face, posture, and context in which the person is involved. Thus, a new classification model that integrates all these characteristics to have more veracity in detecting the feeling will be developed. Once this extension is done, we could make another comparison to Megatron-LM to put in perspective how much value these new features add to our framework. In addition, the framework could be part of an autonomous navigation system applied to a social robot, which would complement decision-making in these processes as well as assuming better socially acceptable behavior towards humans.

## Figures and Tables

**Table 1:** Examples of tagged text in the dataset.

Text	Target
Has anyone else had a bad experience with Poun...	[1, 0, 0, 1, 0, 0, 0, 0, 1, 0, 0]
#America finding #gratitude amidst the sadness...	[0, 0, 0, 1, 0, 0, 1, 0, 0, 0, 0]
Let us not burden our remembrances with a heav...	[0, 0, 0, 0, 0, 0, 1, 0, 1, 0, 0]
@AdsByFlaherty And you’re cheerfully defending...	[1, 0, 1, 0, 0, 0, 0, 0, 0, 0, 0]
The irony and hilarity of making Taylor Lautne...	[0, 0, 0, 0, 1, 0, 0, 0, 1, 0, 0]

**Table 2:** Number of occurrences of the tested emotions in the samples of the training dataset.

Anger	Anticipation	Disgust	Fear	Joy	Love	Optimism	Pessimism	Sadness	Surprise	Trust
1101	425	1099	485	1442	516	1143	375	960	170	153

**Table 3:** Overall results of the the model on the 2018-E-c-En-test-gold dataset.

Average Type	F1 Score	Precision	Recall	Accuracy
Micro	0.664	0.761	0.589	0.535
Macro	0.481	0.650	0.432	

**Table 4:** Tabulated results of the confusion matrix for each emotion. We show the percentage of true negatives (TN), false positives (FP), false negatives (FN), and true positives (TP) per label.

	Anger	Anticip.	Disgust	Fear	Joy	Love	Opt.	Pessim.	Sadness	Surprise	Trust
TN	58.852	85.947	58.515	83.431	50.967	80.853	55.784	86.683	63.025	94.692	95.121
FP	7.364	1.013	7.763	1.688	4.787	3.314	9.144	1.810	7.518	0.092	0.184
FN	9.481	12.059	9.604	6.935	10.954	8.315	10.341	9.880	12.010	5.002	4.664
TP	24.310	0.982	24.118	7.947	33.292	7.518	24.732	1.626	17.367	0.216	0.031

**Table 5:** Comparison of our model with NVIDIA-AI.

Model	Accuracy	Micro F1	Macro F1
Stacked Ensemble (ours)	0.535	0.664	0.481
NVIDIA-AI	0.577	0.690	0.561

## Data Availability

Data available in a publicly accessible repository.
